# Effectiveness and methods of cryotherapy in reducing swelling after total knee arthroplasty: A systematic review on randomized controlled trials

**DOI:** 10.1002/nop2.1906

**Published:** 2023-06-19

**Authors:** Yoonyoung Lee, Yong Soon Shin, Hyun Jung Kim, Jiwon An

**Affiliations:** ^1^ Department of Nursing, Sunchon National University Jeonnam Korea; ^2^ Hanyang University Seongdong‐gu Korea; ^3^ Asan Medical Center Songpa‐gu Korea; ^4^ Far East University Eumseong‐gun Korea

**Keywords:** cryotherapy, evidence‐based practice, intervention research, nursing practice, postoperative nursing, swelling, systematic reviews, total knee arthroplasty

## Abstract

**Aim:**

This study aims to investigate the effect and methods of cryotherapy in reducing swelling after total knee arthroplasty.

**Design:**

Systematic review.

**Methods:**

We searched PubMed, Embase, CINAHL, Cochrane Library, KoreaMed, KERIS and National Science Digital Library for randomized controlled trials on 19 August 2021. This systematic review was conducted according to the PRISMA 2009 checklist.

**Results:**

A total of eight randomized controlled trials were systematically reviewed to determine the effect and methods of cryotherapy on reducing postoperative swelling. The effects were not significantly different in six studies. Application time per cryotherapy session was 10–20 min when using an ice pack and up to 48 h when using an automated device. The duration ranged from 2 days to 1 week or until discharge, and the frequency varied from 2 to 72 times per day.

## 1 INTRODUCTION

Total knee arthroplasty (TKA) is a surgical procedure performed at the final stage of severe knee injury or disability (Kasmire et al., 2014). However, during acute postoperative recovery, patients experience clinical symptoms such as swelling, pain, bleeding and reduced range of motion (Adie et al., 2012). Local swelling after TKA occurs because of tissue damage and inflammatory responses at the surgical site (Thienpont, 2014).

Non‐pharmacological treatments that reduce swelling after TKA include cryotherapy (Thacoor & Sandiford, 2019), multilayer compression therapy (Stocker et al., 2018) and manual lymphatic drainage (Pichonnaz et al., 2016). Cryotherapy is the application of a cold substance to the skin around inflamed soft tissues and joints (Thacoor & Sandiford, 2019). It reduces tissue swelling by decreasing vasospasm and blood flow (Abramson et al., 1966). Basic cryotherapy is performed using a gel pack, ice pack and crushed ice in plastic bags (Thacoor & Sandiford, 2019; Zhong et al., 2021). Advanced cryotherapy involves circulating ice water, the electronic control of continuous cryotherapy and cryopneumatic devices (Thacoor & Sandiford, 2019).

## 2 BACKGROUND

Research on cryotherapy has been conducted since the 1960s (Abramson et al., 1966); studies on cryotherapy application on patients after orthopaedic surgery have started recently (Zhong et al., 2021). However, studies on the effectiveness of cryotherapy in reducing postoperative swelling after TKA are insufficient. Three studies were reported in a comprehensive review that confirmed the effect of cryotherapy on swelling after TKA. The results suggested that the effect on swelling was not consistent (Thacoor & Sandiford, 2019). Moreover, the studies that confirmed the effect of swelling after TKA were not randomized controlled studies (Borgers et al., 2020; Karaduman et al., 2019; Ruffilli et al., 2017; Schinsky et al., 2016). Some studies did not confirm the effect of swelling (Nishigami et al., 2019); two studies compared cryotherapy using ice or gel pack with that using a device (Coviello et al., 2022; Healy et al., 1994).

This study aims to conduct a systematic review of the effectiveness and methods of cryotherapy in reducing postoperative swelling in patients after TKA so that nurses can provide intervention based on scientific evidence in clinical nursing practice.

## 3 METHODOLOGY

A systematic review was performed according to the Preferential Reporting Items for Systematic Reviews and Meta‐Analyses (PRISMA) guidelines (Moher et al., 2015) to identify the effects and methods of cryotherapy on swelling in patients after TKA. This systematic review was registered in PROSPERO on 29 September 2021 (CRD42021274912).

### 3.1 Selection criteria

The key question considered in the systematic literature review was ‘Does cryotherapy help in reducing postoperative swelling in patients after TKA?’ The study was conducted following the population, intervention, comparison, outcome and study design (PICOSD) structure. The inclusion criteria were as follows: the population (P) comprised adult patients over 18 years of age who underwent TKA; the interventions (I) were cryotherapy (gel packs, ice packs, crushed ice in plastic bags, circulating ice water prompting cryotherapy, electronic control of continuous cryotherapy or cryopneumatic devices); comparison (C) with usual or the same type of care as the intervention group excluding cryotherapy; the outcome (O) was knee circumference; and the study design (SD) was all prospective randomized controlled trials (RCTs). We searched the literature for studies with human participants published until 19 August 2021, and included the ones published in all languages.

The exclusion criteria were as follows: (1) non‐TKA surgery, animal subjects; (2) non‐localized cryotherapy, cold irrigation, gaseous cryotherapy, studies using the same type of cryotherapy in all subjects; and (3) non‐RCTs, non‐original articles, case studies, protocols, commentaries, systematic reviews, comprehensive reviews, cohort studies and conference abstracts.

### 3.2 Search strategy and data extraction criteria

The search strategy for the systematic review was developed and conducted by a librarian, an expert in literature search and who had experience in conducting systematic reviews, along with inputs form the authors of this study. On 19 August 2021, the search was conducted using the following electronic databases: PubMed, Cumulative Index to Nursing and Allied Health Literature (CINAHL), Embase (Elsevier platform), Cochrane Central Register of Controlled Trials (Wiley platform), KoreaMed (abstract database of the Korean Association of Medical Journal Editors), Korea Education and Research Information Service (KERIS) and National Science Digital Science Library (NSDL). ClinicalTrials.gov was also searched for ongoing and recently completed trials. The search terms included cryotherapy and swelling. The following terms were used: (‘cryotherapy’ OR ‘cold therapy’ OR ‘ice’ OR ‘local cooling’) AND (‘edema’ OR ‘swelling’) AND (‘knee joint/surgery’ OR ‘arthroplasty, replacement, knee’ OR ‘TKA’ OR ‘TKR’). We used a combination of keywords and indexed terms (e.g. Medical Subject Headings). The search results were exported to EndNoteX8 (Clarivate Analytics), and duplicate articles were excluded.

Two researchers independently evaluated the search results. After reviewing the title and abstract, the selected studies underwent a full‐text review. Disagreements between the researchers were addressed through discussion and, if necessary, a third researcher's evaluation.

### 3.3 Data extraction

The first researcher extracted data from the studies included in this research and the second confirmed their accuracy. Disagreements between the two were addressed through discussion. The data extracted from each selected study included general characteristics (first author, publication year and country), study participants (sample size), intervention methods (material/device, application site, temperature, application time, frequency, application time per time and application duration), control condition and outcome (outcome, time points of measurements and results of swelling).

### 3.4 Assessing the risk of bias

Version 2 of the Cochrane Collaboration's risk of bias tool (RoB 2; Higgins et al., 2019) was used to assess the quality of the selected RCTs. For each selected study, two researchers extracted and confirmed information across five domains: the randomization process, deviations from the intended interventions, missing outcome data, measurement of the outcome and selection of the reported result. Based on the RoB 2 results, the full text of each article was identified as exhibiting high risk, some concerns or low risk. Two reviewers independently evaluated the articles and discussed differences to reach a consensus.

## 4 RESULTS

### 4.1 Selected studies

**FIGURE 1:**
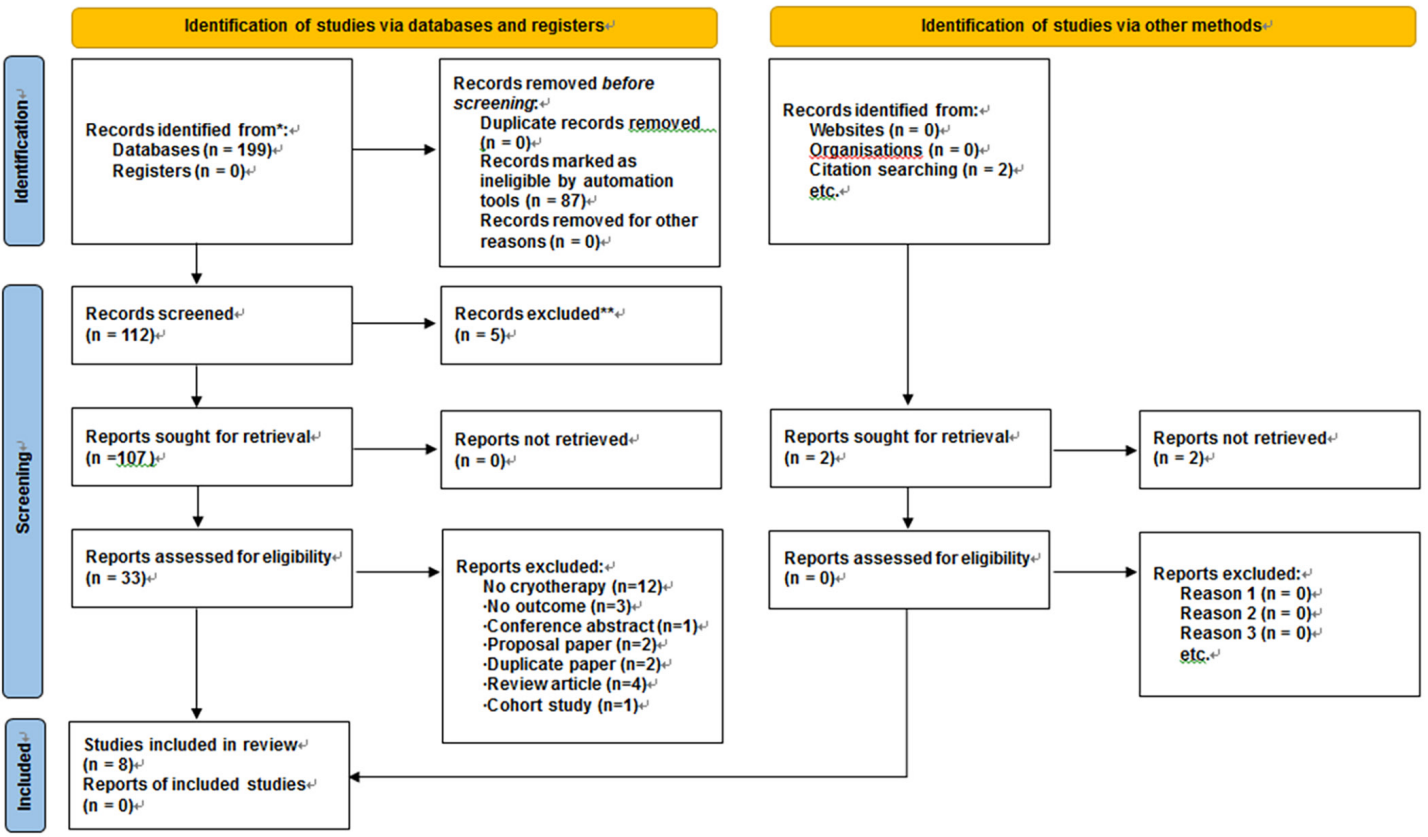
PRISMA flow diagram.

The study selection process for this systematic review is shown in Figure 1. Because of searching the six databases, 199 articles (55 in PubMed, 67 in Embase, 29 in the Cochrane Library, 38 in CINAHL, one in KoreaMed, seven in KERIS and two in NSDL) were identified. Additionally, 201 articles were identified by searching two grey literature databases. After removing 94 duplicate studies, the titles and abstracts of 107 articles were checked, and 33 that met the inclusion criteria were selected. Among these, 25 articles were excluded: 12 non‐cryotherapy studies, three non‐swelling studies, one conference abstract without full text, two proposal papers, two duplicate published studies, four review articles and one cohort study. Finally, eight were included in the systematic review.

### 4.2 Study characteristics

The characteristics of the eight studies included in this systematic review are summarized in Table 1. Among them, five studies were published within the last 5 years (Brouwers et al., 2022; Chen et al., 2020; Stocker et al., 2018; Thijs et al., 2019; Wang, 2017) and three were published more than 5 years ago (Hecht et al., 1983; Holmström & Härdin, 2005; Webb et al., 1998). The studies were based in the Netherlands (Brouwers et al., 2022; Thijs et al., 2019), Taiwan (Chen et al., 2020), the United States (Hecht et al., 1983), Sweden (Holmström & Härdin, 2005), Switzerland (Stocker et al., 2018), China (Wang, 2017) and the United Kingdom (Webb et al., 1998). There were 490 participants across all studies; their number per study ranged from 16 to 106.

**TABLE 1:** Descriptive summary of the included studies.

	First author Publication year Country	Patients	Intervention 1. Material or device 2. Site 3. Temperature 4. Time per session 5. Frequency 6. Duration	Control condition	Outcome	Site of measurements	Time points of measurements	Results about swelling
1	Brouwers 2022 The Netherlands	102 patients Int (*n* = 51) Cont (*n* = 51)	1. Computer‐assisted cryotherapy Brace type 2. Around the knee 3. 6–10°C 4. 2–6 h 5. Continuous 6. During the first seven postoperative days	Usual care	*Primary outcome* Swelling *Secondary outcome* Pain, use of opioid escape medication, AROM and patient satisfaction	Mid‐patella, 7 cm proximally of the patella 7 cm distally of the patella	2 weeks postoperatively 6 weeks postoperatively	No significant difference Proximal *p* = 0.532 Int: 46.0 (6.0) Cont: 45.3 (5.8) Mid‐patellar *p* = 0.768 Int: 43.7 (4.1) Cont: 43.4 (4.3) Distal *p* = 0.293 Int: 38.9 (4.8) Cont: 39.9 (4.8)
2	Chen 2020 Taiwan	60 patients Int (*n* = 30) Cont (*n* = 30)	1. Cryotherapy pack and CPM 2. No information 3. No information 4. 20 min 5. Intermittent (continued for 20 min and then stopped for 30 min) 6. Started using a CPM machine and applied programmed cryotherapy intermittently within 1 h while returning to the ward on the day of surgery From POD 1 to the day of discharge	CPM Received cryotherapy on POD 1 and the application of cryotherapy was not programed	*Primary outcome* Swelling *Secondary outcome* ROM, pain	15 cm proximal to the superior pole of the patella 15 cm distal to the inferior pole of the patella	POD 1 POD 4 or until the patients were discharged	No significant difference *p* = 0.157 Int: M33.2 Cont:M33.9
3	Hecht 1983 USA	36 patients Int 1 (*n* = 13) Int 2 (*n* = 13) Cont (*n* = 10)	Int1: Local cold application with exercise therapy 1. Cold packs 2. Anterior and posterior areas of the knee 3. No information 4. 20 min 5. Once before knee exercise 6. No information Int 2: Heat with exercise therapy 1. Hot packs (four layers of towelling) 2. Anterior, medial and posterior areas of the knee 3. No information 4. 20 min 5. No information 6. No information	Exercise therapy	*Primary outcome* Swelling *Secondary outcome* ROM, pain	5 cm above superior patellar border mid‐patella 5 cm below inferior patellar border	After the first session of knee exercise After the 10th session of knee exercise	**After the 10th session of knee exercise** 5 cm above superior patellar border No significant difference Int 1: −1.00 (0.54) Int 2: −0.09 (0.33) Cont: −0.49 (0.37) Mid‐patella *p* < 0.05 Int 1: −1.43 (0.30) Int 2: 0.58 (0.47) Cont: −0.43 (0.40) 5 cm below inferior patellar border No significant difference Int1: −1.27 (0.87) Int2: −0.30 (0.55) Cont: −0.20 (0.38)
4	Holmström 2005 Sweden	61 patients Int 1 (*n* = 23) Int 2 (*n* = 21) Cont (*n* = 17)	Int 1 1. Cold therapy combined with hydrostatic pressure 2. Front of the knee 3. 10–15°C 4. 48 h 5. Continuous 6. Immediately after skin closure and before the release of the tourniquet ~48 h Int 2: Epidural anaesthesia	Traditional analgesics	*Primary outcome* Swelling *Secondary outcome* Pain, bleeding, range of motion, function and morphine consumption	Mid‐patella	POD 7 6 weeks	No significant difference
5	Stocker 2018 Switzerland	16 patients Int (*n* = 8) Cont (*n* = 8)	1. Standard treatment with an ice pack Physiopack 13 × 30 cm 2. Knee 3. No information 4. 10 min 5. 3 times per day 6. POD 1–POD 5	Multi‐layer compression therapy	*Primary outcome* Swelling *Secondary outcome* ROM, pain and function	5 cm proximal to the joint space 10 cm proximal to the joint space 15 cm distal to the joint space	POD 1 POD 3 POD 6 6 weeks	**POD 3** 10 cm proximal *p =* 0.02 −1.8 cm (95%CI: −3.2; −0.5) **POD 6** 5 cm proximal *p* < 0.001 −2.7 (95%CI: −4.1; −1.3) 10 cm proximal *p* < 0.001 −3.8 (95%CI: −5.1; −2.3) Joint space *p* = 0.01 −2.7 (95%CI: −4.6; −0.7) 15 cm distal *p* = 0.01 −2.1 (95%CI: −3.6; −0.6) **6 weeks** 5 cm proximal *p* = 0.01 −2.2 (95%CI: −3.6; −0.8) 10 cm proximal *p* < 0.001 −2.5 (95%CI: −3.9; −1.1)
6	Thijs 2019 The Netherlands	60 patients Int (*n* = 30) Cont (*n* = 30)	1. Computer‐assisted cryotherapy (CAC) 2. Knee 3. 10–12°C 4. Received 6 h of continuous cooling 5. 2 h of cryotherapy in the morning, followed by 2 h in the afternoon 6. Immediately after surgery‐POD 7	Computer‐assisted cryotherapy (CAC) 21°C Received 6 h of continuous cooling	*Primary outcome* Swelling *Secondary outcome* Pain, consumption of opioid and visual haematoma	Mid‐patellar 7 cm distally 7 cm proximally	Pre‐ 1 weeks 2 weeks 6 weeks	No significant difference
7	Wang 2017 China	106 patients Int (*n* = 53) Cont (*n* = 53)	1. Local compression cryotherapy combined with continuous passive motion 2. Knee (570 × 360 mm) 3. 11–13°C 4. Continuous 5. Continuous 6. After arrival at ward—48 h	Continuous passive motion	*Primary outcome* Swelling *Secondary outcome* Pain	2 cm proximal patella	24 h 48 h	**24 h** *p* < 0.001 Int: 0.42 (0.11) Cont: 0.75 (0.18) **48 h** *p =* 0.006 Int: 0.72 (0.24) Cont: 1.20 (0.32)
8	Webb 1998 United Kingdom	49 patients Int (*n* = 24) Cont (*n* = 25)	1. Cold therapy combined with hydrostatic pressure (Cryo/Cuff, 30 mmHg) 2. Front of the knee 3. No information 4. No information 5. Continuous 6. No information	Wool and crepe dressing (compression)	*Primary outcome* Swelling *Secondary outcome* Blood loss, pain and range of motion	2 cm proximal to the patella	Pre POD 5 6 week 3 months	No significant difference

### 4.3 Risk of bias assessment

**FIGURE 2:**
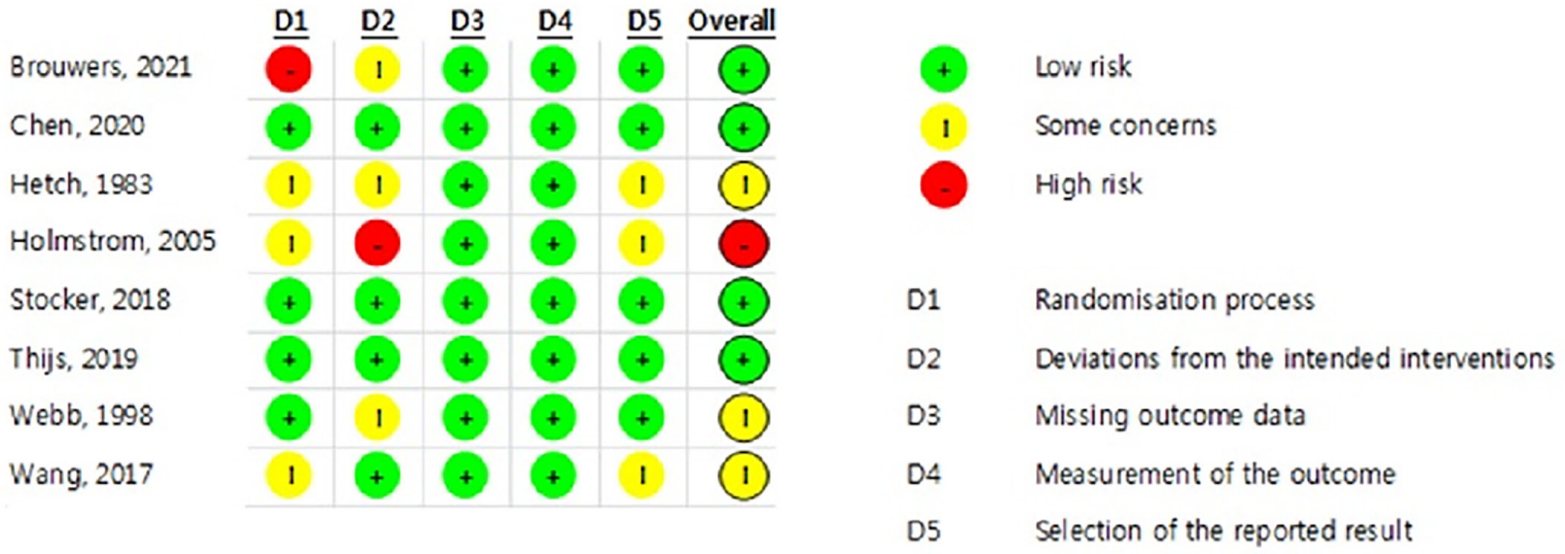
Risk of bias result.

**FIGURE 3:**
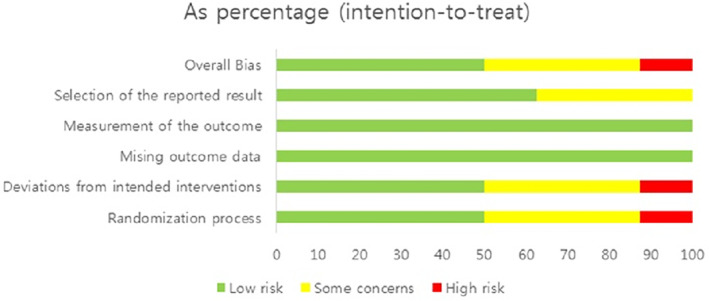
Risk of bias summary.

The quality assessment results of the eight selected studies are presented in Figures 2 and 3. Regarding overall bias, four studies had a low risk (Brouwers et al., 2022; Chen et al., 2020; Stocker et al., 2018; Thijs et al., 2019), three had some concerns (Hecht et al., 1983; Wang, 2017; Webb et al., 1998), and one had a high risk (Holmström & Härdin, 2005).

### 4.4 Intervention and outcome measures

Among the eight selected studies, five used computer‐assisted cryotherapy material or device to apply cryotherapy (Brouwers et al., 2022; Holmström & Härdin, 2005; Thijs et al., 2019; Wang, 2017; Webb et al., 1998); ice pack was used in the remaining three studies (Chen et al., 2020; Hecht et al., 1983; Stocker et al., 2018).

Regarding the application site, cryotherapy was applied to the front of the knee in four studies (Holmström & Härdin, 2005; Stocker et al., 2018; Wang, 2017; Webb et al., 1998), around the knee in one (Brouwers et al., 2022) and to the anterior and posterior knees in another (Hecht et al., 1983). Two studies did not state the application site (Chen et al., 2020; Thijs et al., 2019).

The application temperature of cryotherapy was 10–15°C in one study (Holmström & Härdin, 2005), 10–12°C in another (Thijs et al., 2019). It was 11–13°C in the study by Wang (2017), and 6–10°C in that by Brouwers et al. (2022). Four studies did not provide the applied temperature (Chen et al., 2020; Hecht et al., 1983; Stocker et al., 2018; Webb et al., 1998).

The application time per session was 10 min in Stocker et al. (2018). It was 20 min for Chen et al. (2020) and Hecht et al. (1983), 2–6 h for Brouwers et al. (2022), 6 h for Thijs et al. (2019), 48 h for Holmström and Härdin (2005) and Wang (2017). One study did not provide the application time per session (Webb et al., 1998). Application frequency of cryotherapy was continuous for four studies (Brouwers et al., 2022; Holmström & Härdin, 2005; Wang, 2017; Webb et al., 1998). One applied cryotherapy 72 times per day (Chen et al., 2020), another applied it 10 times (Hecht et al., 1983). One of them applied it three times per day (Stocker et al., 2018), and another two times per day (Thijs et al., 2019).

Regarding the start time of cryotherapy after TKA, three studies (Holmström & Härdin, 2005; Thijs et al., 2019; Wang, 2017) started the application on the operation day, and three (Brouwers et al., 2022; Chen et al., 2020; Stocker et al., 2018) on the first postoperative day. Two studies did not provide the start time (Hecht et al., 1983; Webb et al., 1998).

Regarding the application duration of cryotherapy, one study applied it from the first to the seventh postoperative day (Brouwers et al., 2022), one from the first postoperative day to discharge (Chen et al., 2020), one from skin closure to 48 h (Holmström & Härdin, 2005), one from the first postoperative day to the fifth postoperative day (Stocker et al., 2018), one from after surgery to the seventh postoperative day (Thijs et al., 2019) and one from ward to 48 h (Wang, 2017). Two studies did not provide the application duration (Hecht et al., 1983; Webb et al., 1998).

The time points of measurement were the first postoperative day (Chen et al., 2020; Stocker et al., 2018; Wang, 2017), second postoperative day (Wang, 2017), third postoperative day (Stocker et al., 2018), fourth postoperative day (Chen et al., 2020), fifth postoperative day (Stocker et al., 2018) and sixth postoperative day (Stocker et al., 2018). It was first postoperative week (Holmström & Härdin, 2005; Thijs et al., 2019), second postoperative week (Brouwers et al., 2022; Thijs et al., 2019), sixth postoperative week (Brouwers et al., 2022; Holmström & Härdin, 2005; Stocker et al., 2018; Thijs et al., 2019; Webb et al., 1998), third postoperative month (Webb et al., 1998) and discharge (Chen et al., 2020).

The effects of cryotherapy were not significantly different in six studies (Brouwers et al., 2022; Chen et al., 2020; Hecht et al., 1983; Holmström & Härdin, 2005; Thijs et al., 2019; Webb et al., 1998). However, in one, the swelling decreased in the cryotherapy group compared to the control group after 24 and 48 h (Wang, 2017). In another study, swelling decreased in the cryotherapy group the third and sixth postoperative day and sixth postoperative week compared to the control group (Stocker et al., 2018).

## 5 DISCUSSION

This study aimed to examine the existing RCT literature on the effectiveness and methods of cryotherapy in reducing postoperative swelling after TKA to enable nurses to provide scientific interventions in clinical nursing practice.

Recently published studies that confirmed the reduction in postoperative swelling in patients after TKA were comprehensive reviews. One was on the orthopaedic application of cryotherapy (Kunkle et al., 2021), and another was to check the evidence of cryotherapy applied to TKA (Thacoor & Sandiford, 2019). Moreover, a systematic review of cryotherapy after TKA published 10 years ago for RCT studies was based on blood loss, visual analogue score (VAS) pain, adverse events, range of motion, transfusion rate, function, analgesia use, swelling, length of hospital stay, the effect on the quality of life and activity level. However, it did not focus on the effect of postoperative swelling (Adie et al., 2012; Ni et al., 2015).

A systematic literature review showed that cryotherapy was effective in reducing swelling after TKA in two studies and had no effect in six studies at any time point. In a previous study, two out of nine studies confirmed a decrease in swelling (Kunkle et al., 2021). Among the comprehensive reviews, one study confirmed the opposite result according to the ice water exchange time, another had a conflict of interest with the cryotherapy device manufacturer, and another measured only 25% of the study subjects and found no effect on the reduction of swelling (Thacoor & Sandiford, 2019). Therefore, this study's results were similar to those of previous studies, and the effect on swelling after TKA surgery could not be confirmed.

The application temperature of cryotherapy to reduce swelling after TKA was confirmed in only four cases (50%). In three studies, a temperature of 10–15°C was applied; it was 6–10°C in another. Theoretical evidence has suggested that the effect on pain reduction can be confirmed when the skin surface temperature is 13.5°C or less after the application of cryotherapy (Bugaj, 1975; Chesterton et al., 2002). However, scientific evidence for the application of temperature to reduce swelling after TKA has not been confirmed. Therefore, further studies should be conducted regarding this.

The application times per cryotherapy session to reduce swelling after TKA varied and were 10 min, 20 min, 2–6 h, 6 h and 48 h. The application within 20 min was in the case of using local ice pack or device for more than 2 h. This is similar to the study result when cryotherapy was applied for musculoskeletal problems and the duration per session was 10–20 min (Shin et al., 2018). In computer‐assisted cryotherapy, it was applied from 2 to 48 h. This was different from a previous study, wherein required the use of a device to prevent continuous cryotherapy application below 65°F for longer than 2 h (Brown & Hahn, 2009). Since the application time per session of cryotherapy varies, further studies considering side effects are needed.

Regarding the application site of cryotherapy after TKA, six studies applied it to the front of the knee, and two to the front and back. The standard incision for TKA is a middle line incision (Sanna et al., 2013). Therefore, in most studies, cryotherapy was applied to the front of the knee. While applying materials or devices, the back of the knee seemed to be the convenient site for application. However, such an application site may increase discomfort, and therefore, should be selected according to the subject's comfort.

The frequency of cryotherapy application varied from at least twice a day to continuous application. When a device capable of maintaining a constant temperature was used, it could be used continuously. Conversely, a material (ice pack) that can cause temperature changes and tissue damage can be frequently applied to maintain cryotherapy effect and prevent side effects. Therefore, further research is needed to confirm the most effective application frequency for reducing swelling after TKA to prevent side effects as per various application methods.

Regarding the start time of cryotherapy after TKA, three studies started the application on the day of surgery, and three on the first postoperative day. However, a previous study reported that swelling increased by 35% on the first postoperative day (Pua, 2015). Therefore, further studies are needed to confirm whether cryotherapy applied immediately after surgery is more effective than the one applied on the day following the surgery.

The application period of cryotherapy was different for each study—from 2 days to 1 week or after discharge. According to the study results (Gao et al., 2011), swelling after TKA occurred from the day after surgery and lasted for at least a week. Therefore, continuous intervention is necessary to reduce swelling. The long‐term application in clinical practice will significantly affect the nurses' workload; therefore, it is important to understand the scientific evidence.

As a result, five studies (62.5%) applied computer‐assisted cryotherapy and three (37.5%) ice pack cryotherapy. Recently, an increasing number of studies confirmed the effectiveness of automated devices for the continuous application of cryotherapy to damaged tissues (Borgers et al., 2020; Brouwers et al., 2022). Moreover, an advanced RCT study that used a mirabilite with an ice pack was conducted for comparison with a study that used a general ice pack (Zhong et al., 2021). Most studies did not confirm the effects of routine care and cryotherapy but identified new devices or materials compared to the effects of existing cryotherapy. Therefore, the effectiveness of cryotherapy could not be confirmed.

Regarding overall bias, four studies had a low risk, three had some concerns and one had a high risk. The study, evaluated as high‐risk, was conducted in 1983. Since the study was conducted 40 years ago, it does not meet the research design required by the current RCT. Four studies evaluated as low‐risk were well‐designed RCTs within the last 5 years. Therefore, if more well‐designed RCT studies are conducted, the effect of cryotherapy on swelling can be confirmed.

In addition to this study, the following study proposes study a multimodal protocol in the management of swelling after TKA such as study a multimodal protocol for reduction of pain after TKA (Spinarelli et al., 2016).

This study had several limitations. First, despite sufficient search strategies and no language or date restrictions, this review included only a small number of studies. There were 490 participants across all studies, and their number per study ranged from 16 to 106. Second, although RCTs were targeted, there RoB was low in only four out of eight studies.

## 6 CONCLUSION

This study's results showed that the effect and methods of cryotherapy on swelling after TKA could not be confirmed. The application temperature of cryotherapy for pain reduction has been confirmed; however, evidence pointing to any reduction in swelling is lacking. Further studies are required in the backdrop of an increase in the use of automated devices that apply cryotherapy to damaged tissues. To reduce swelling and prevent side effects according to the various application methods, the most effective application time, frequency, site and duration for reducing oedema after TKA should be identified and applied.

## AUTHOR CONTRIBUTIONS

All authors contributed in conception of review question, searching the literatures and conducting all steps of study, and approved the final article.

## FUNDING INFORMATION

This research was registered in PROSPERO (CRD42021272944). This research was funded by a basic research project the Government of Korea (Ministry of Science, ICT and Future Planning) with the support of the Korea Research Foundation grant number (NRF‐2014R1A1A3052149).

## CONFLICT OF INTEREST STATEMENT

The authors declare no conflict of interests.

## ETHICS STATEMENT

This systematic review of reviews used data from previously published reviews; therefore, no ethical approval was required.

## PATIENT OR PUBLIC CONTRIBUTION

Application of cryotherapy on reducing postoperative swelling should be applied differently in time, duration, and frequency based on evidence.

## Data Availability

Data will be made available upon a formal request to the authors.
